# Impacts of hydrous manganese oxide on the retention and lability of dissolved organic matter

**DOI:** 10.1186/s12932-018-0051-x

**Published:** 2018-02-13

**Authors:** Jason W. Stuckey, Christopher Goodwin, Jian Wang, Louis A. Kaplan, Prian Vidal-Esquivel, Thomas P. Beebe, Donald L. Sparks

**Affiliations:** 1 Biology Department, Multnomah University, Portland, OR 97220 USA; 2 Department of Plant & Soil Sciences and Delaware Environmental Institute, University of Delaware, Newark, DE 19716 USA; 3 Department of Chemistry and Biochemistry, University of Delaware, Newark, DE 19716 USA; 4 Canadian Light Source Inc., University of Saskatchewan, Saskatoon, SK S7N 2V3 Canada; 5 Stroud Water Research Center, 970 Spencer Road, Avondale, PA 19311 USA; 6 Delaware Environmental Institute, University of Delaware, Newark, DE 19716 USA

**Keywords:** Soil carbon, Dissolved organic matter, Manganese oxide, Goethite, Organo-mineral associations

## Abstract

Minerals constitute a primary ecosystem control on organic C decomposition in soils, and therefore on greenhouse gas fluxes to the atmosphere. Secondary minerals, in particular, Fe and Al (oxyhydr)oxides—collectively referred to as “oxides” hereafter—are prominent protectors of organic C against microbial decomposition through sorption and complexation reactions. However, the impacts of Mn oxides on organic C retention and lability in soils are poorly understood. Here we show that hydrous Mn oxide (HMO), a poorly crystalline δ-MnO_2_, has a greater maximum sorption capacity for dissolved organic matter (DOM) derived from a deciduous forest composite O_i_, O_e_, and O_a_ horizon leachate (“O horizon leachate” hereafter) than does goethite under acidic (pH 5) conditions. Nonetheless, goethite has a stronger sorption capacity for DOM at low initial C:(Mn or Fe) molar ratios compared to HMO, probably due to ligand exchange with carboxylate groups as revealed by attenuated total reflectance-Fourier transform infrared spectroscopy. X-ray photoelectron spectroscopy and scanning transmission X-ray microscopy–near-edge X-ray absorption fine structure spectroscopy coupled with Mn mass balance calculations reveal that DOM sorption onto HMO induces partial Mn reductive dissolution and Mn reduction of the residual HMO. X-ray photoelectron spectroscopy further shows increasing Mn(II) concentrations are correlated with increasing oxidized C (C=O) content (r = 0.78, P < 0.0006) on the DOM–HMO complexes. We posit that DOM is the more probable reductant of HMO, as Mn(II)-induced HMO dissolution does not alter the Mn speciation of the residual HMO at pH 5. At a lower C loading (2 × 10^2^ μg C m^−2^), DOM desorption—assessed by 0.1 M NaH_2_PO_4_ extraction—is lower for HMO than for goethite, whereas the extent of desorption is the same at a higher C loading (4 × 10^2^ μg C m^−2^). No significant differences are observed in the impacts of HMO and goethite on the biodegradability of the DOM remaining in solution after DOM sorption reaches steady state. Overall, HMO shows a relatively strong capacity to sorb DOM and resist phosphate-induced desorption, but DOM–HMO complexes may be more vulnerable to reductive dissolution than DOM–goethite complexes.

**Electronic supplementary material:**

The online version of this article (10.1186/s12932-018-0051-x) contains supplementary material, which is available to authorized users.

## Introduction

Carbon exchange between the Earth’s surface and atmosphere is a fundamental regulator of the climate system. Historically an increase in atmospheric temperature has accompanied an increase in atmospheric carbon dioxide (CO_2_) concentration [1]. Carbon exchange within terrestrial systems results from photosynthesis, autotrophic respiration, and microbial respiration [2]. Plant-derived organic C enters the soil through leaf and wood detrital decomposition, throughfall and root exudation. Organic C may persist in soils for millennia before returning to the atmosphere as CO_2_ or methane (CH_4_) or being exported to groundwater as dissolved organic carbon (DOC) or dissolved inorganic carbon (DIC) [2]. In fact, the soil C pool is greater than the vegetative and atmospheric C pools combined [3]. Therefore, a process-level understanding of C storage and fluxes within soils is paramount to projecting future climatic conditions.

The growing consensus of the predominant means by which soils store and stabilize C over the long-term is by mineral protection, especially by secondary aluminosilicates and metal oxides [3–9]. Organo-mineral complexes may hinder the efficacy of microbial enzymes to degrade organic C [6, 10, 11]. The major proposed mechanisms of organic C sorption to minerals include anion exchange (electrostatic interaction), ligand exchange-surface complexation, cation bridging, Van der Waals forces, hydrogen bonding, and hydrophobic interactions [5, 12]. Montmorillonite, for instance, exhibits selective sorption of low molecular weight dissolved organic C moieties most probably through a relatively weak cation or water bridging mechanism [13]. Metal oxides, on the other hand, may have a greater capacity to sorb C on a mass basis (mg C g^−1^) than aluminosilicates do resulting from a higher specific surface area [10, 14, 15]. Further, the importance of Fe and Al oxides relative to silicate minerals in stabilizing C generally increases with increased soil development [16, 17]. Iron oxides are often the most prominent minerals stabilizing organic C in soils [9, 10, 18–20]. Goethite, for instance, has a strong affinity for DOM through a ligand exchange reaction resulting in Fe-carboxylate bonds on the goethite surface [13, 15]. In acidic forest soils, Al oxides play a particularly important role in protecting organic C against microbial degradation through the formation of organo-hydroxy-Al complexes during organic litter decomposition, and potentially by Al toxicity to microbes [8, 21–23]. Aluminum oxide-DOM complexes may leach into the subsoil (B and C horizons), promoting long-term C storage [10].

Manganese oxides represent a third class of metal oxides that plays a complex and salient role in the cycling of C within soils and the forest floor [13, 23–25]. Manganese oxides may be enriched in organic C relative to the bulk soil [26], and poorly crystalline δ-MnO_2_ in particular, can serve as a significant reservoir of organic C in terrestrial environments [27]. In forest ecosystems, the rate of aboveground plant litter decomposition regulates partitioning of organic C into soil organic matter and CO_2_ [28]. Factors controlling the plant litter decomposition rate include temperature, moisture, litter quality (e.g., lignin content), and resource availability (e.g., DOC, nutrients, and Mn) to the decomposer community [24, 29–37]. Manganese is present initially as Mn(II) in live foliage and becomes enriched through the litter decomposition process due to carbon loss [29, 38, 39]. Fungi accumulate and oxidize Mn(II) to Mn(III), which in turn promotes the oxidative decomposition of litter, regenerating the Mn(II) [24]. Fungi reoxidize Mn(II) to the Mn(III) through the early stages of decomposition [24]. The Mn(III) is likely temporarily stabilized in solution by chelating ligands [24, 40]. In later stages of decomposition, Mn partitions to Mn(III/IV) oxides [24, 41–43].

Manganese oxide-induced organic C oxidation may cause decomposition to more labile substrates and ultimately to CO_2_ [13, 23, 25, 44]. Manganese oxides may oxidize organic acids, such as pyruvate, but not other acids, such as formate and lactate (at least in the timescale of hours) [44–46]. The oxidative potential of Mn oxides translates into enhanced microbial decomposition of non-cellulosic polysaccharides, but not of cellulosic polysaccharides or lignin [23, 25]. Thus, the impact of the complex redox chemistry occurring between Mn oxides and DOM on the partitioning of C to CO_2_ and organic compounds of varying complexity and oxidation state remains poorly defined. Further, the impacts of Mn oxides on the lability of DOM, and therefore our ability to predict C exchange between soils—with ubiquitous Mn oxides—and the atmosphere remains elusive.

Much of the work on the interactions between Mn oxides and organic matter has been performed on model organic compounds [47–51], alkaline extracts (i.e., humic substances) [44, 52, 53], or under alkaline conditions [54]. We are aware of one other study that has studied the extent and mechanism of water-extracted natural DOM sorption to a Mn oxide (i.e., birnessite), showing a low sorption capacity relative to goethite and reductive dissolution of birnessite coupled with oxidative transformation of the DOM through an adsorption mechanism, though the C moieties involved in the surface complexation and oxidation of DOM are not clear [13]. The extent and mechanism of water-extracted natural DOM sorption onto HMO—a poorly crystalline δ-MnO_2_ analogous to vernadite and a Mn oxide more closely related to biogenic Mn oxides than is birnessite—has not been studied. Nor have the impacts of HMO on the chemical lability and biological degradability of water-extracted natural DOM been examined.

Accordingly, the objectives of this study are to assess the impacts of HMO on the retention, chemical lability, and biological degradability of DOM (as present in an O horizon leachate) from a deciduous forest soil. Here we use goethite as a positive control in our experiments, as the impacts of goethite on the cycling of forest floor-derived DOM are relatively well established [13, 15, 19, 55]. We hypothesize first that HMO will have a lower DOM sorption capacity than goethite due to a lower point of zero charge; secondly, DOM sorbed to HMO will be more labile than that sorbed to goethite, as Mn oxides are stronger oxidants than Fe oxides, and therefore HMO may reductively dissolve in the presence of DOM; thirdly, the greater oxidative capacity of HMO will increase the biodegradability of DOM remaining in solution post-reaction with HMO compared to that reacted with goethite or to the initial, pre-reacted DOM. Here we employ batch sorption and desorption experiments, bioreactor systems, and state-of-the-art analytical techniques including XPS, ATR-FTIR, and synchrotron STXM–NEXAFS, to elucidate the reactions occurring between DOM and the respective metal oxides.

## Methods

### O horizon leachate and mineral preparation and characterization

The O horizon leachate was obtained through a water extraction of the O horizon (O_i_, O_e_, and O_a_; approximately 2 cm thick) of an Ultisol under a deciduous forest at the Stroud Water Research Center in Avondale, PA predominantly consisting of tulip poplar (*Liriodendron tulipifera*), American beech (*Fagus gradifolia*), red maple (*Acer rubrum*), and red oak (*Quercus rubra*). The extraction mass ratio was 1:2 (1 kg field moist litter: 2 kg DI water), and the suspension was shaken for 90 h in the dark on an end-to-end rotary shaker at 200 rpm, exhibiting a pH of 4.5. The O horizon leachate was passed through a 2 mm sieve to remove coarse particulates and centrifuged at 20,000*g* for 2 h. The supernatant was vacuum filtered successively through 0.8, 0.45 and 0.22 μm polyethersulfone filters.

The O horizon leachate total Mn, Fe, Cu, Zn, Al, Ca, Mg, K and Na content was determined by ICP–OES (Thermo Elemental Intrepid II XSP Duo View, Waltham, MA, USA). Dissolved Fe(II) was measured by the 1,10-phenanthroline method [56], and Mn speciation was assessed qualitatively in a freeze-dried sample using XPS (Thermo scientific K-alpha^+^ XPS, East Grinstead, United Kingdom). Total organic C, total C, and total N were measured using a TOC Analyzer (Elementar Americas Vario Mx CN, Mt. Laurel, NJ, USA).

Hydrous Mn oxide (poorly crystalline δ-MnO_2_), a Mn(IV) oxide similar to biogenic Mn oxides [57], and goethite were synthesized by standard methods and maintained as concentrated suspensions [58–60]. Briefly, HMO was synthesized by drop wise addition of 0.15 M Mn(NO_3_)_2_·4H_2_O to a solution comprised of 0.1 M KMnO_4_ and 0.2 M NaOH. The resulting suspension was stirred overnight (at least 12 h) to allow complete conproportionation of Mn(II) and Mn(VII) to Mn(IV), and the HMO was used for all experimentation within 3 weeks of synthesis [61]. Goethite was made by slow (~ 48 h) oxidation of dissolved FeCl_2_ buffered to pH 7 by NaHCO_3_. The identity and purity of the minerals were confirmed by XRD (Additional file 1: Figure S1). The specific surface area of the minerals was determined by the BET equation applied to N_2_ adsorption data acquired at 77 K for relative pressures of 0.05 to 0.3 with a Micromeritics ASAP 2020 surface area analyzer (Norcross, GA, USA) [62, 63]. Particle size and electrophoretic mobility were measured simultaneously in deionized water by dynamic light scattering (Wyatt Technologies Möbiuζ, Santa Barbara, CA), resulting in a calculation of zeta potential using the DYNAMICS software package (Wyatt Technologies). The point of zero charge (PZC) for HMO and goethite used in this study is 1.9 and 8.0, respectively [15, 64].

### Sorption experiment

Sorption of DOM (from the O horizon leachate) onto HMO and goethite was performed at 22 °C over initial molar C:(Mn or Fe) ratios of 0.2–9 by reacting 45 mg (dry weight equivalent) of mineral (HMO/goethite) suspensions with 45 ml of leachate solution of DOC varying concentration, yielding a solid:solution ratio of ~ 1:1000 g dry wt mL^−1^. The initial C:(Mn or Fe) molar ratios are derived from the DOC concentration of the leachate—equivalent to the total C concentration within error—and the initial solid-phase Mn or Fe concentration. The pH of the suspensions was maintained at 5.0 ± 0.2 by addition of HCl or NaOH. The total volume of HCl and/or NaOH required to achieve and maintain a pH of 5.0 ± 0.2 was ≤ 1% of the total initial suspension volume. The suspensions were shaken in the dark on an end-to-end rotary shaker at 150 rpm for 24 h, which was adequate time for steady state to be achieved (Additional file 1: Figure S2). Subsequently, the suspensions were centrifuged at 20,000*g* for 30 min. The settled material was washed twice with DI water to remove the remainder of the equilibrium solution before freeze-drying [63]. Total C of the freeze-dried mineral-DOM complexes was measured using a vario Micro cube CHNS Analyzer (Elementar Analysensysteme GmbH, Langenselbold, Germany).

### Desorption experiment

Desorption of DOM from the sorption complexes was performed by reacting the moist solid-phase products with 10 mL of fresh 0.1 M NaH_2_PO_4_ (pH 4.5) for two sequential 24 h periods as described previously [63], with one modification of increased shaking speed to 150 rpm. The centrifuged (20,000*g*) supernatants from the two extraction steps were combined and filtered with a 0.45 μm filter and acidified to 1% HCl (trace metal grade) for total Mn or Fe analysis by microwave plasma-atomic emission spectroscopy (Agilent Technologies 4100 MP-AES, Santa Clara, CA).

### Biodegradation of non-sorbed DOM

Biofilm reactors colonized and sustained by a continual perfusion with White Clay Creek stream water containing DOM and suspended bacteria were used to measure the aerobically biodegradable dissolved organic carbon (BDOC) content of leaf litter leachates as described previously [65]. White Clay Creek is the stream adjacent to the site where the composite O horizon sample was collected to prepare leachate that was then reacted with HMO and goethite at an initial C:(Mn or Fe) molar ratio of 3.1. The BDOC of pre- and post-reaction leachates were measured. Details of the bioreactor design and methods for determining BDOC are provided in the Supplementary Material.

### X-ray photoelectron spectroscopy

The XPS measurements were taken at the University of Delaware’s Surface Analysis Facility (SAF) using a Thermo scientific K-alpha^+^ XPS (East Grinstead, United Kingdom). Monochromatic aluminum K-alpha x-rays where used with a spot size of 100 μm, the flood gun was used to limit charging effects. Each sample had a survey spectrum taken with a 100 eV pass energy and 1 eV step. High-resolution scans were performed for every element found in any sample with atomic percent greater than 0.1%, and the pass energy and step size used were 20 eV and 0.1 eV, respectively. The powder samples where mounted on carbon tape with care to limit contamination. The pressed powders were hundreds of μm thick and the photoelectron escape depth is in the nm scale [66], and therefore the carbon tape did not contribute to any of the XPS spectra. To determine sample homogeneity and reproducibility, duplicate measurements where taken on each sample, and the results show that for elements found with greater than 2 atomic percent the signal variance was 1.5%—well within the accepted range of 5% [67].

All peak processing was done in CasaXPS version 2.3.16. The following C types were distinguished: C bonded to C or H (C–C, C=C, C–H; at 284.6 eV), C singly bonded to O or N (C–O, C–N; at 286.1 eV), and C with multiple bonds to O (C=O, O–C–O; at 288.0 eV) similar to previous XPS analysis on DOM [68, 69]. The carbon spectra were fit with a Shirley background and due to the amount of organic and inorganic material, 70-30 Gaussian–Lorentzian mix peaks were used with no constraints on peak position or peak broadness (Additional file 1: Figure S3). All full width half max values did not vary between species or sample by more than 0.2 eV, and the peak position did not vary by more than 0.2 eV. Manganese (Mn 2p 3/2) and Fe 2p 3/2 spectra were fit in a similar way to [70] with the position and width of the peaks constrained to the Mn(IV), Mn(III), Mn(II), Fe(III), and Fe(II) standards. Standards used for Mn and Fe XPS fitting were Mn(II) oxide (Sigma-Aldrich, CAS Number: 1344-43-0), Mn(III) oxide (Sigma-Aldrich, CAS Number: 1317-34-6), Mn(IV) oxide (Sigma-Aldrich, CAS Number: 1313-13-9), Fe(III) oxide (Sigma-Aldrich, CAS Number: 1309-37-1), and Fe(II)Cl_2_ (Sigma-Aldrich, CAS Number: 13478-10-9).

### Attenuated total reflectance-Fourier transform infrared spectroscopy

The ATR-FTIR spectra were collected with a Bruker Tensor 27 FTIR spectrometer (Billerica, MA, USA) using the standard Pike ATR cell. Samples were freeze-dried and scanned over a range of 4000–600 cm^−1^ with a 2 cm^−1^ resolution. An average spectrum was obtained from 128 scans for each sample with the OPUS Data Collection Program (Version 7.2) (Bruker Corporation), and baseline subtraction was performed with GRAMS/AI Spectroscopy Software (Version 9.2) (Thermo Fisher Scientific, Inc.). To obtain a spectrum of DOM associated with HMO or goethite, the baseline-corrected spectrum of pure HMO or pure goethite was subtracted from the spectrum of the DOM–HMO or DOM–goethite complex, respectively. Spectra were not normalized as all DOM peaks were impacted by the sorption reaction. Therefore, comparisons between ATR-FTIR spectra were limited to peak position and relative ratios of peak intensities.

### Scanning transmission X-ray microscopy

In order to examine the spatial distribution and speciation of DOM sorbed onto HMO and goethite, STXM–NEXAFS was performed at the C K-edge, N K-edge, metal (Mn or Fe) L-edge on DOM–HMO and DOM–goethite sorption complexes at beamline 10ID-1 at the Canadian Light Source as described previously for DOM-ferrihydrite complexes [63]. The DOM–HMO and DOM–goethite complexes were analyzed at two C loadings each: 128 ± 3.1 μg m^−2^ (“low”) and 428 ± 29 μg m^−2^ (“high”) for HMO and 207 ± 0.4 μg m^−2^ (“low”) and 406 ± 6.9 μg m^−2^ (“high”) for goethite. The elemental detection limit for STXM–NEXAFS was ~ 0.1% [71]. The aXis2000 software package was used for image and spectra processing [72]. Linear combination fitting of Mn L-edge STXM–NEXAFS spectra was optimized over a range of 635–660 eV using four reference spectra of Mn oxide standards of varying oxidation state [73]. Linear combination of Fe L-edge STXM–NEXAFS spectra was optimized over a range of 700–730 eV using FeO and Fe_2_O_3_ reference spectra from the authors’ own database. A 1 nm thick elemental X-ray absorption profile was calculated with known chemical composition and density for each reference compound; each reference spectrum was scaled to its elemental X-ray absorption profile to obtain a reference spectrum of 1 nm thickness, which was used for the linear combination fitting [74]. The contribution of each standard to the linear combination fit (in nm) was converted to a weight % using the standard’s density.

## Results

### Mineral and O horizon leachate characterization

Hydrous Mn oxide is less crystalline than goethite and has two characteristic peaks at 37° and 66° 2θ (Cu Kα) (Additional file 1: Figure S1) [75]. The N_2_-BET specific surface area (SSA) values obtained for the HMO and goethite are virtually equivalent—138.0 ± 1.3 m^2^ g^−1^ and 140.0 ± 1.8 m^2^ g^−1^, respectively—and are comparable to those found elsewhere (Table 1) [15, 57, 76]. The mean particle diameter is in the sub-micron range and has a unimodal distribution for both HMO and goethite. The O horizon leachate has a wider particle size distribution, and shows evidence of flocculation in solution after filtration, as the mean particle diameter is greater than 0.2 μm (Table 1). Hydrous Mn oxide and O horizon leachate are both negatively charged, whereas goethite is positively charged (Table 1).

**Table 1:** Characterization of hydrous Mn oxide, goethite, and leaf litter leachate (pH 4.5)

Material	Specific surface area (m^2^/g)	Mean particle diameter (nm)	Zeta potential (mV)	Electrophoretic mobility (μm/s[V/cm]^−1^)
Hydrous Mn oxide	138 ± 1.3	309 ± 16	− 502 ± 46	− 502 ± 46
Goethite	140 ± 1.8	661 ± 81	+ 284 ± 36	+ 284 ± 36
Dissolved NOM	N/A	429 ± 170	− 223 ± 68	− 223 ± 68

Error bars indicate standard deviation of mean for triplicate measurements

The leachate has a pH of 4.5 and electrical conductivity of 0.156 S m^−1^ (Table 2). The C:N molar ratio is 10.5, and previous characterization of the leachate from the site showed that 36% of the total N is present at NH_4_^+^ and 0.05% is present as NO_3_^−^ (data not shown). Dissolved Mn in the leachate is predominantly Mn(II) (Additional file 1: Figure S4), and ~ 40% of the aqueous Fe is present as Fe(II) (data not shown), suggesting a substantial presence of complexed Fe(III) in solution. The dissolved Mn:Fe molar ratio is 13.1 in the leachate. In deciduous (e.g. maple) foliage, Mn:Fe molar ratios may range from 7.2 to 100 [77]. The O horizon leachate contains a high Ca level (2.5 mM), which may promote DOM sorption to metal oxides [78, 79].

**Table 2:** Chemical composition of leaf litter leachate

pH	Electrical conductivity (S/m)	TOC (mg L^−1^)	Total C (mg L^−1^)	Total N (mg L^−1^)	Mn (μM)	Fe (μM)	Cu (μM)	Zn (μM)	Al (μM)	Ca (μM)	Mg (μM)	K (μM)	Na (μM)
4.5 ± 0.0	0.156 ± 0.00	1893 ± 11.8	1894 ± 11.3	210 ± 2.6	854 ± 4.2	65.4 ± 0.9	7.0 ± 1.7	16 ± 0.4	185 ± 5.5	2543 ± 45	1720 ± 49	2079 ± 40	188 ± 12

Error bars indicate standard deviation of mean for triplicate measurements

### Organic C speciation of DOM and DOM-mineral complexes

**Fig. 1:**
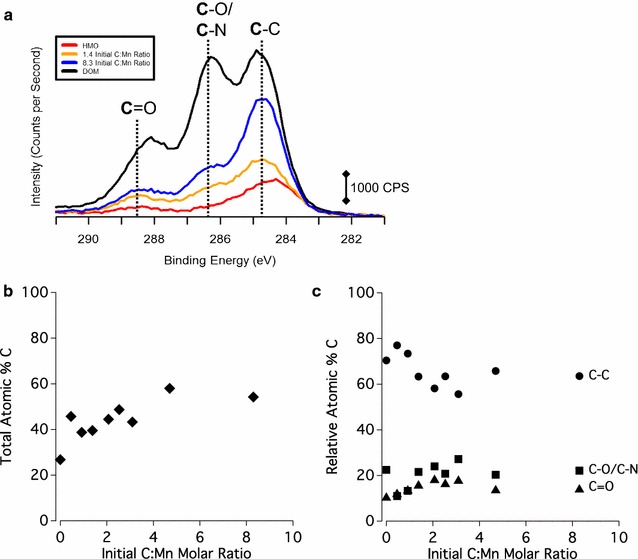
**a** XPS of the C 1s region of the DOM shown in black, 8.3 initial C:Mn molar ratio samples in blue, 1.4 initial C:Mn molar ratio sample in yellow, and untreated HMO shown in red. The region is broken into three distinct species: the most oxidized is the C that is double bonded to O labeled as C=O at 288.2 eV, the middle species is labeled as C–O and C–N at 286.1 eV, and the least oxidized carbon is the C–C or C–H carbon at 284.6 eV. **b** The C atomic percent of each sample based on every element detected with XPS. **c** The most reduced C species, the C–O and C–N associated C, and the most oxidized C (C=O), each expressed by relative atomic percent of the total C signal as a function of the initial C:Mn molar ratio

**Fig. 2:**
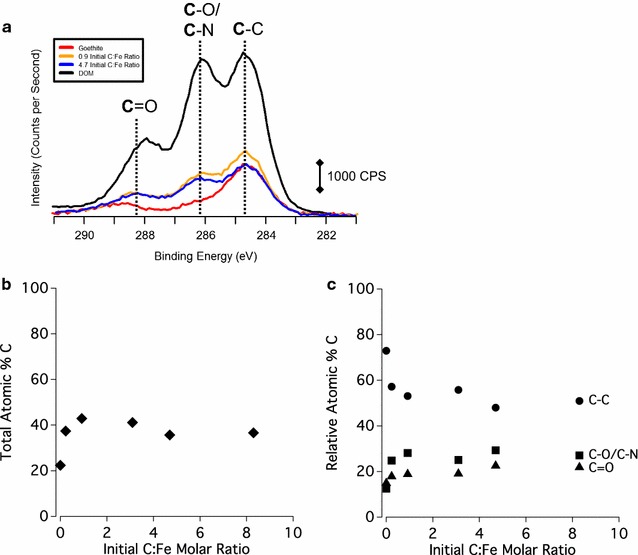
**a** XPS of the C 1s region of the DOM shown in black, 4.7 initial C:Fe molar ratio samples in blue, 0.9 initial C:Fe molar ratio sample in yellow, and untreated goethite shown in red. The region is broken into three distinct species: the most oxidized is the C that is double bonded to O labeled as C=O at 288.2 eV, the middle species is labeled as C–O and C–N at 286.1 eV, and the least oxidized carbon is the C–C or C–H carbon at 284.6 eV. **b** The C atomic percent of each sample based on every element detected with XPS. **c** The most reduced C species, the C–O and C–N associated C, and the most oxidized C (C=O), each expressed by relative atomic percent of the total C signal as a function of the initial C:Mn molar ratio

The C 1s XPS spectrum of the initial (unreacted) DOM contains 3 main C peaks: the most reduced (C–C) C peak, which includes reduced moieties, as well the adventitious C adsorbed from the air [80, 81], the C–O/C–N peak chiefly indicative of polysaccharides and/or amino acids [68, 69, 82] and the oxidized (C=O) C peak (Figs. 1, 2). The unreacted HMO shows evidence of primarily adventitious C (Fig. 1a), and the goethite contains adventitious C as well as a small oxidized C peak likely from residual oxidized carbon associated with the goethite synthesis procedure (Fig. 2a). All three C peaks in the unreacted DOM are present in the C 1s XPS spectra of the DOM–HMO and DOM–goethite complexes. Increasing C loading on HMO and goethite shows a decrease and subsequent stabilization in the percent carbon signal of reduced (C–C) C, and an increase and subsequent leveling off of the percent carbon signal of both the polysaccharide/amino acid-associated C (C-O and C-N) and the oxidized (C=O) C (Figs. 1c and 2c).

**Fig. 3:**
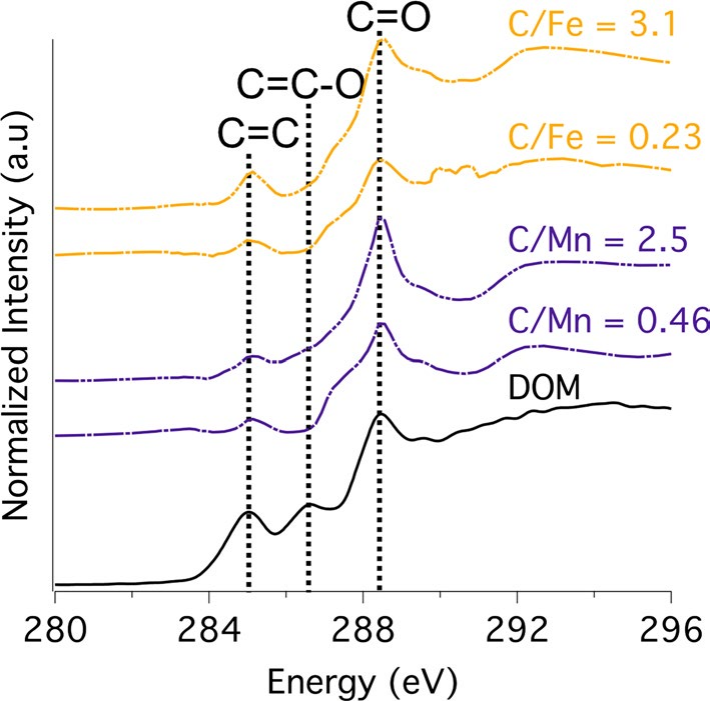
Carbon 1s NEXAFS spectra collected at a synchrotron-based scanning transmission X-ray microprobe for the unreacted DOM, HMO with initial C:Mn molar ratios of 0.46 and 2.5, and goethite with initial C:Fe molar ratios of 0.23 and 3.1. The aromatic (C=C), phenolic (C=C–O), and carboxylic (C=O) C peaks locations are shown for reference

The C NEXAFS spectrum of the unreacted DOM has three main peaks: an aromatic (π*_C=C_) peak at 285.1 eV, a phenolic (π*_C=C–O_) peak at 286.5 eV, and a prominent carboxylic (π*_C=O_) peak at 288.4 eV as obtained previously (Fig. 3) [63]. Sorption of the DOM onto the HMO and goethite results in a dampening of the aromatic C peak and a disappearance of the phenolic C peak with the carboxylic peak remaining pronounced (Fig. 3). Increasing C loading onto the HMO and goethite results in an increase in the carboxylic C peak intensity.

**Fig. 4:**
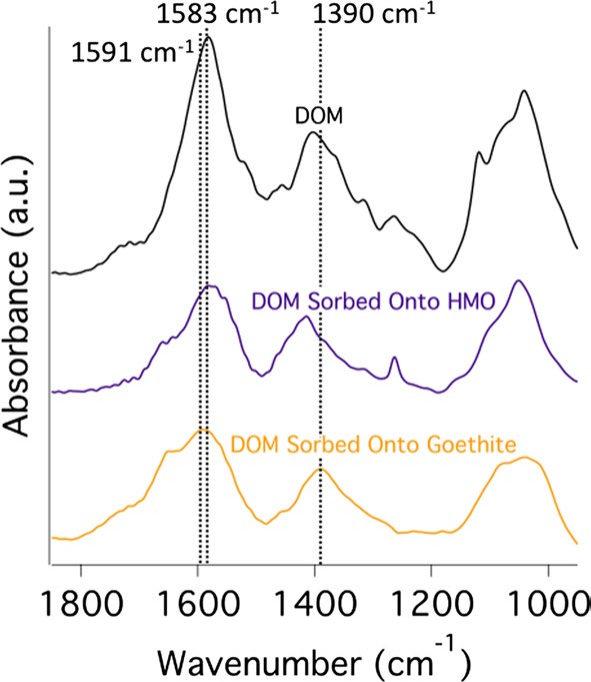
ATR-FTIR spectra of DOM–HMO and DOM–goethite sorption complexes in comparison with that of the unreacted DOM. The DOM–HMO and DOM–goethite sorption complexes result from initial C:metal molar ratios of 3.1 and have comparable C loadings (59.6 ± 7.1 mg C g^−1^ and 56.8 ± 1.0 mg C g^−1^, respectively)

The ATR-FTIR spectrum of the unreacted DOM shows predominant peaks at 1583 and 1404 cm^−1^ indicative of an asymmetric COO^−^ stretch and symmetric COO^−^ stretch, respectively, as well as a peak at 1043 cm^−1^ representing a C–O stretch of polysaccharides (Fig. 4; Additional file 1: Table S1). Sorption of DOM onto goethite shifts the asymmetric COO^−^ peak from 1583 to 1591 cm^−1^ and shifts the symmetric COO^−^ peak from 1404 to 1390 cm^−1^—indicative of carboxylate-metal bond formation – and decreases the symmetric COO^−^ peak/C–O stretch of polysaccharides (at ~ 1042 cm^−1^) ratio from 1.27 to 1.18 (Fig. 4). Sorption of DOM onto HMO does not shift the asymmetric COO^−^ peak (providing no indication of carboxylate-metal bond formation), shifts the symmetric COO^−^ peak from 1404 to 1414 cm^−1^, shifts the predominant C–O stretch of polysaccharides from 1043 to 1051 cm^−1^, and decreases the symmetric COO^−^ peak/C–O stretch of polysaccharides ratio from 1.27 to 0.95 (Fig. 4).

### Nanoscale spatial distribution of DOM on HMO and goethite

**Fig. 5:**
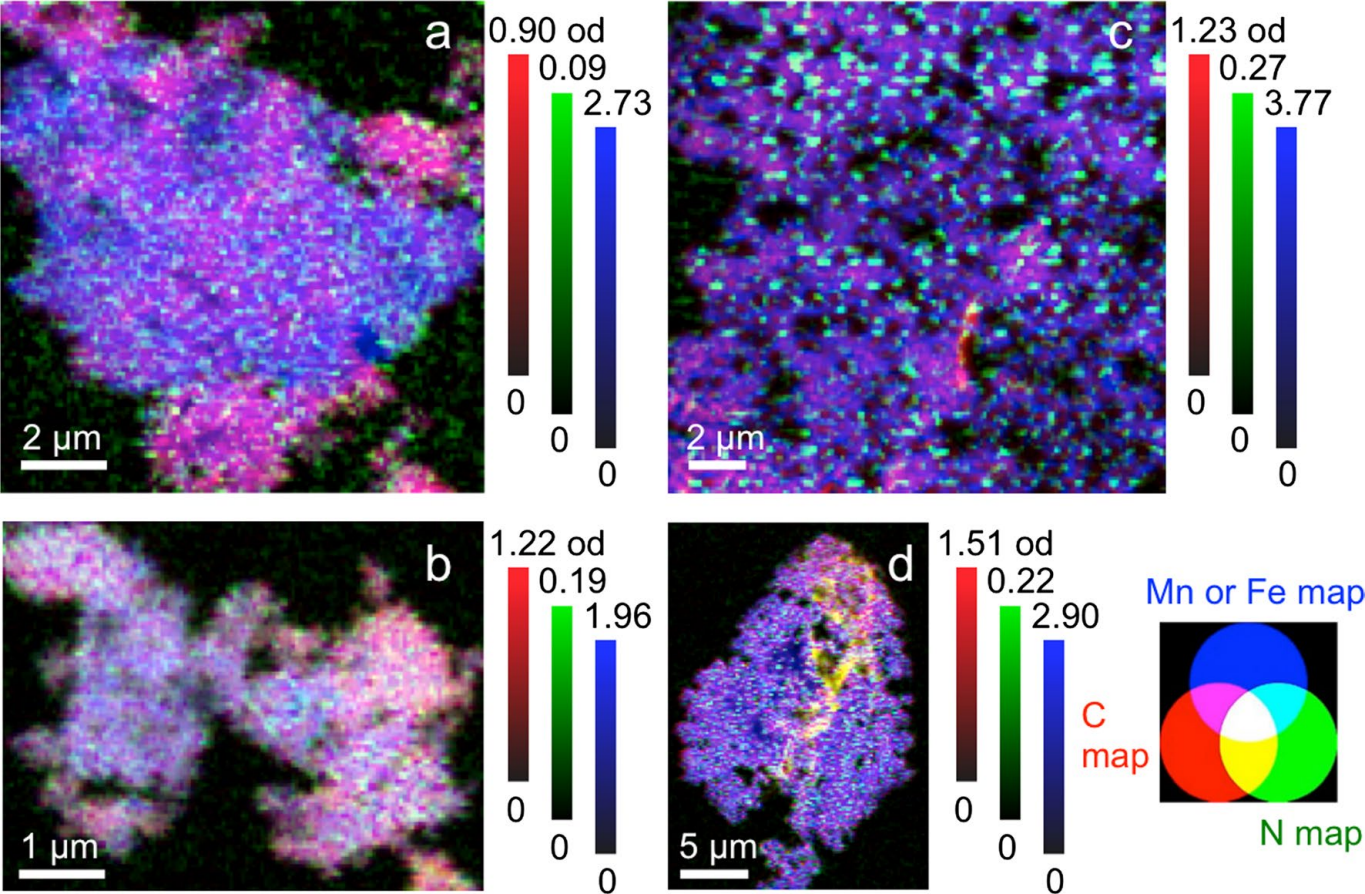
Color-coded composite STXM maps of C (red), N (green), and metal (blue; Mn for HMO and Fe for goethite) for **a** HMO with initial C:Mn ratio of 0.46, **b** HMO with initial C:Mn ratio of 2.5, **c** goethite with initial C:Fe ratio of 0.23, and **d** goethite with initial C:Fe ratio of 3.1. Color bars are optical density ranges for each element in each specific sample

Heterogeneity of C distribution decreases with increasing C loading on the HMO (Fig. 5). Carbon hotspots occur at the low C loading, and C is more homogenously distributed at the high C loading. No distinct C phases are observed irrespective of C loading on the HMO. Nitrogen is homogeneously distributed at both low and high C loadings.

Carbon hotspots occur at both low and high C loadings on the goethite, but C is more homogeneously distributed at the high C loading (Fig. 5). No distinct C phases are observed irrespective of C loading on the goethite. Nitrogen is homogeneously distributed at both low and high C loadings.

### Sorption of DOM and quantifying mineral dissolution

**Fig. 6:**
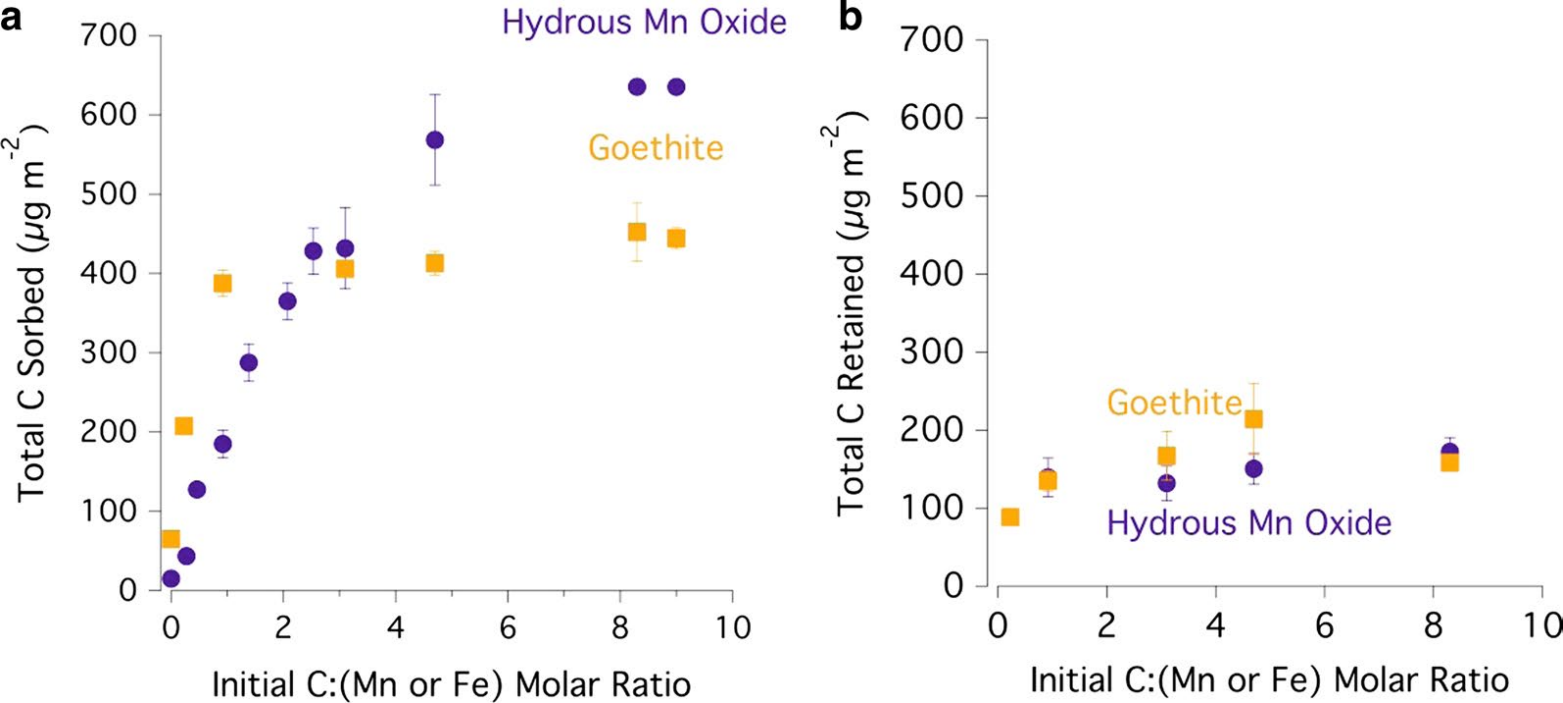
**a** Total C sorbed (normalized by specific surface area) onto hydrous Mn oxide (HMO) and goethite as a function of initial C to metal molar ratio in the batch system. The C:Mn molar ratio reflects the initial moles of C in DOC and moles of Mn in the HMO present. The C:Fe molar ratio reflects the initial moles of C in DOC and moles of Fe in the goethite present. **b** The total C retained on HMO and goethite after extraction with 0.1 M NaH_2_PO_4_ as a function of initial C:(Mn or Fe) molar ratio. Error bars indicate standard deviations of triplicates

Goethite shows a sharp increase in sorption of organic C up to 388 μg C m^−2^ for low (~ < 1) initial C:Fe molar ratios, with slight increases in organic C sorption up to 478 μg C m^−2^ for higher initial C:Fe molar ratios (Fig. 6). Hydrous Mn oxide has a lower affinity for organic C at low (~ < 1) initial C:Mn molar ratios, but has a higher C sorption capacity at higher initial C:Mn molar ratios, retaining 635 μg C m^−2^ for an initial C:Mn molar ratio of 9. The increase in total atomic percent C as detected by C 1s XPS as a function of initial C:metal molar ratio corroborates the C sorption trend observed using the CHNS analyzer (Figs. 1b, 2b, 6).

Reaction of HMO and goethite with DI water for 24 h results in 3.7 μM Mn and 9.1 μM Fe in solution, respectively (Additional file 1: Figure S5; Initial C:(Mn or Fe) ratio = 0), indicating negligible mineral dissolution or metal desorption from the solid phase. However, HMO and goethite show differential stability upon reaction with the O horizon leachate. The HMO batch system shows increasing Mn release into solution—and HMO dissolution—with increasing initial C:Mn molar ratio (Additional file 1: Figure S5). The net change in dissolved Mn in the goethite system, as well as in the dissolved Fe in both the HMO and goethite systems is negative, indicating a net re-partitioning of dissolved metals to the solid phase upon reaction with the O horizon leachate. Thus, contrary to HMO, no leachate-induced dissolution of goethite is observed.

The electrical conductivity of the leachate solutions reacted with HMO and goethite ranges from 5.7 × 10^−3^ to 1.5 × 10^−1^ S m^−1^. Adopting a pseudo-linear relationship between electrical conductivity and ionic strength [83], the ionic strength of the leachate solutions ranges from approximately 0.8–24 mM. Ionic strength variance has negligible impact on the adsorption of DOM onto mineral oxide surfaces for freshwater solutions with an ionic strength less than 100 mM [84].

### Manganese reduction of HMO by O horizon leachate

**Fig. 7:**
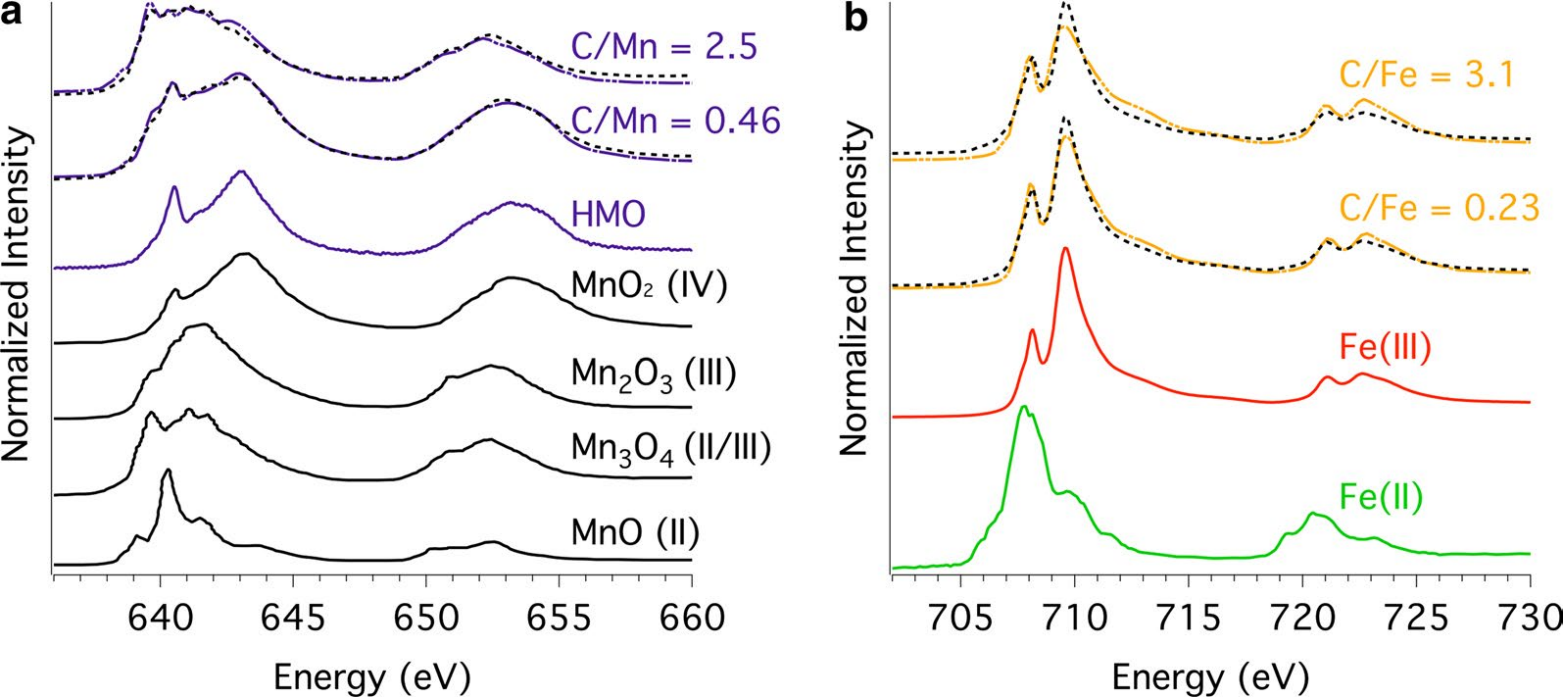
**a** Scanning transmission X-ray microscopy-Mn L-edge NEXAFS spectra for the DOM–HMO complexes with initial C:Mn molar ratios of 0.46 and 2.5 (solid lines), as well as the linear combination fits (dashed lines). The unreacted HMO spectrum and reference spectra for Mn oxide standards from Gilbert, Frazer [73] are provided for comparison. **b** Scanning transmission X-ray microscopy-Fe L-edge NEXAFS spectra for goethite with initial C:Fe molar ratios of 0.23 and 3.1 Iron(II) and Fe(III) reference spectra are shown for comparison

**Fig. 8:**
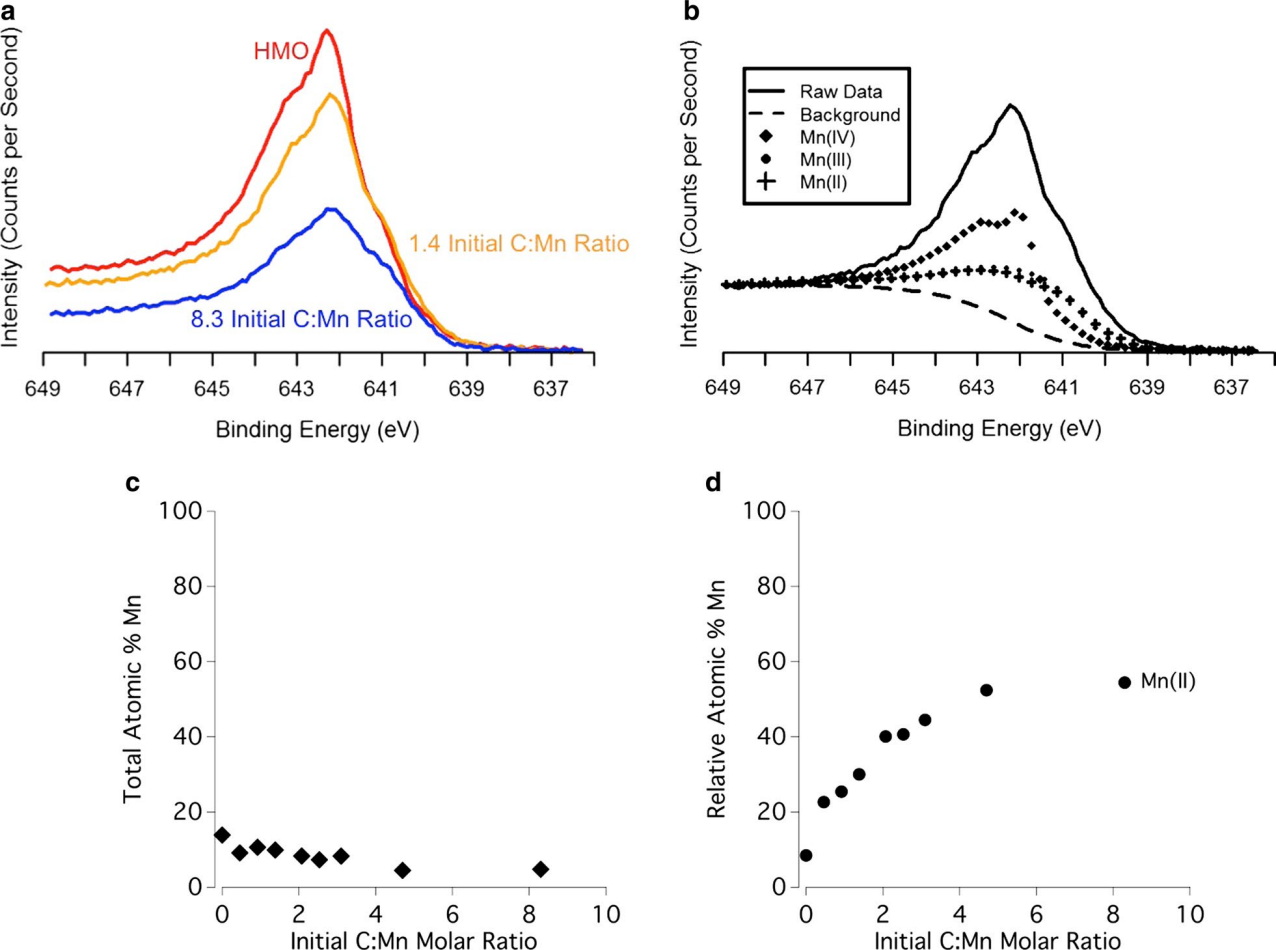
**a** XPS of the Mn 2p 3/2 region of the 8.3 initial C:Mn molar ratio samples in blue, 1.4 initial C:Mn molar ratio sample in yellow, and untreated HMO shown in red. The region is broken into two distinct species; the one of lower binding energy located at 640.7 eV is the Mn(II) peak, and the higher energy peak is a custom line shape of the untreated HMO. **b** The raw Mn XPS spectrum along with the background and Mn XPS standard spectra used for fitting. **c** The Mn atomic percent based on all of elements detected with XPS. **d** The increase in the % of Mn present as Mn(II) as the samples were exposed to increasing amounts of DOM

Scanning transmission X-ray microscopy-Mn L-edge NEXAFS shows that the unreacted HMO is predominantly in the form of Mn(IV) in accordance with other studies (Fig. 7a) [57, 61]. Reaction of HMO with increasing O horizon leachate concentration results in increasing Mn reduction of the HMO (Fig. 7a). For instance, as initial C:Mn molar ratio increases from 0.46 to 2.5, the proportion of MnO_2_ in the resulting DOM–HMO complex decreases from 64% (w/w) to 10% (w/w), whereas the proportion of Mn(II/III) oxides increases from 36% (w/w) to 90% (w/w) (Additional file 1: Table S2). Congruently, Mn XPS shows an increasing proportion of Mn(II) with increasing C loading onto the HMO (Fig. 8d). For instance, as initial C:Mn molar ratio increases from 0.46 to 8.3, the percent of the total Mn present as Mn(II) increases from 23 to 54% (Fig. 8d). An increase in the Mn(II) concentration in the DOM–HMO sorption complexes is strongly correlated with an increase in the oxidized (C=O) C atomic % (r = 0.78, P < 0.0006) (Additional file 1: Table S3).

**Fig. 9:**
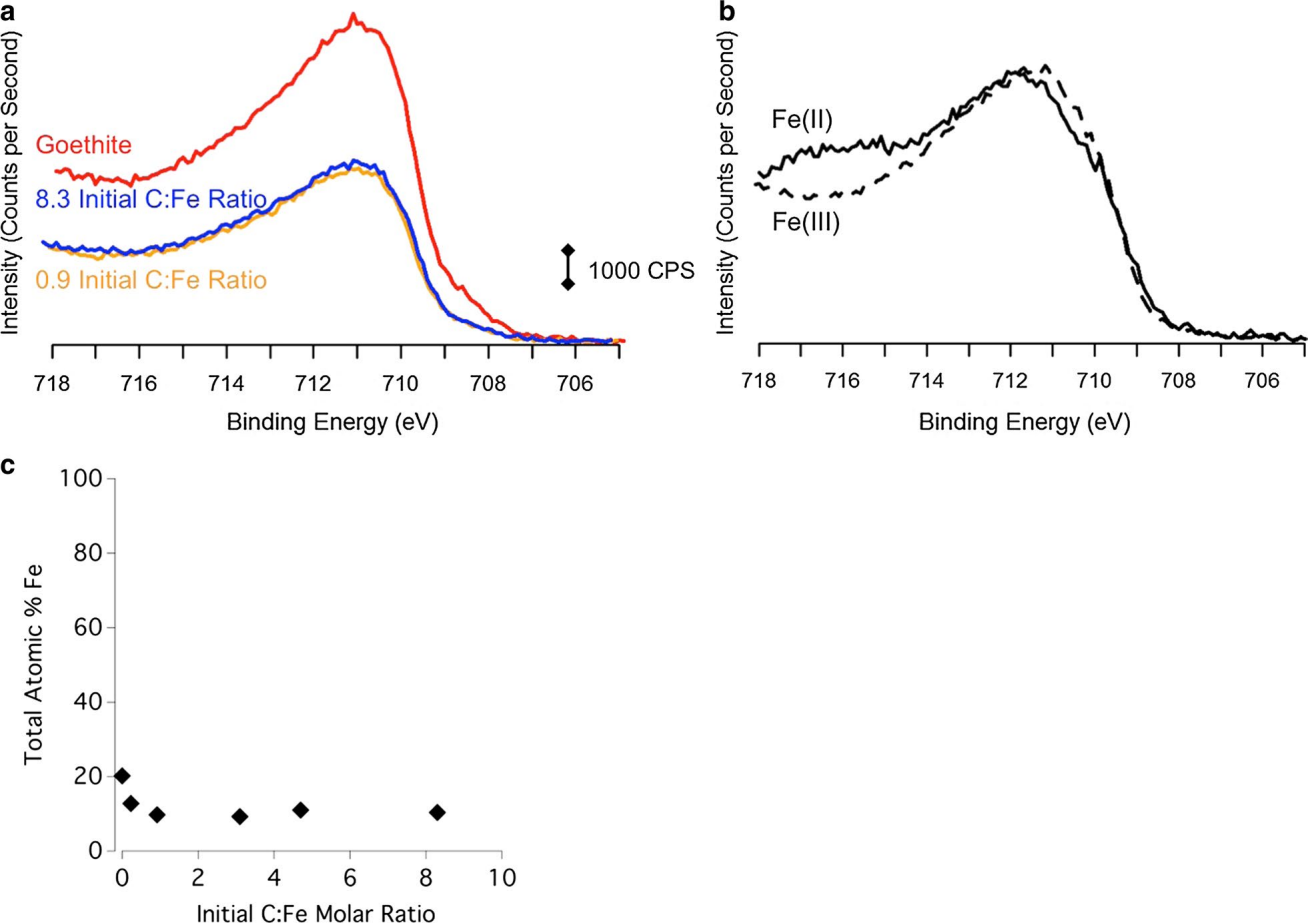
**a** XPS of the Fe 2p 3/2 region of 8.3 initial C:Fe molar ratio sample in blue, 0.9 initial molar ratio sample in yellow, and untreated goethite shown in red. The region shows no major change as DOM loading is increased. **b** The Fe XPS spectra of the Fe(II) and Fe(III) standards used to fit the goethite Fe XPS spectrum. **c** The Fe atomic percent as a function of increasing initial C:Fe molar ratio

On the other hand, sorption of DOM onto goethite induces a relatively low extent of Fe(III) reduction in the STXM-Fe L-edge NEXAFS spectra (Fig. 7b). For instance, as initial C:Fe molar ratio increases from 0.23 to 3.1, the proportion of FeO in the resulting DOM–goethite complex increases from 10% (w/w) to 18% (w/w) (Additional file 1: Table S4). According to Fe XPS, a surface-sensitive technique, Fe(II) is below quantifiable detection in the DOM–goethite complexes (Fig. 9).

### Desorption of DOM

Oxyanions (e.g., H_2_PO_4_^−^ and SO_4_^2−^) are known to compete with DOM for binding sites on metal oxide surfaces, resulting in DOM release to solution [85, 86]. For instance, H_2_PO_4_^−^ forms strong bonds on metal oxide surfaces via surface complexation [87]. Total P and total S are ~ 0.6 and 0.4% of the total C in the O horizon leachate on a molar basis (data not shown), respectively, and therefore H_2_PO_4_^−^ and SO_4_^2−^ most probably provide minimal competition with DOM for sorption sites in this batch system. However, adding H_2_PO_4_^−^ in excess—in the form of a 0.1 M NaH_2_PO_4_ extraction—may serve as an estimate for the amount of DOM capable of being desorbed through ligand exchange [15]. At a low (1.9 × 10^2^–2.1 × 10^2^ C μg m^−2^) loading range, the mean percent of C desorbed from HMO and goethite by extraction with 0.1 M NaH_2_PO_4_ is 25 ± 16% (w/w) and 57 ± 4%, respectively (Fig. 1). At higher C loadings, the mean percent of C desorbed increases in the HMO system, ranging from 69 ± 15 to 74 ± 13%, and remains roughly constant relative to the lower C loading in the goethite system, ranging from 48 ± 7 to 67 ± 2% (Fig. 1).

Reaction with 0.1 M NaH_2_PO_4_ results in release of 2.5–2.8% (mol-basis) of the initial solid-phase Mn of HMO into the aqueous phase and 0.1–0.2% (mol-basis) of the initial solid-phase Fe of goethite into the aqueous phase (Additional file 1: Figure S6). The high release of desorbed Mn from HMO is attributable to O horizon leachate-induced dissolution of HMO and Mn introduced with the O horizon leachate, whereas the low desorbed Fe levels in the goethite system are corroborative evidence for the lack of observed goethite dissolution. The pH of 0.1 M NaH_2_PO_4_ is 4.5, and therefore and Mn or Fe released into solution should not result from acidity-induced dissolution of HMO and goethite, as the minerals are stable under even more acidic conditions [4, 57]. The 0.1 M NaH_2_PO_4_ extraction performed on the initial HMO and initial goethite resulted in 0.08% of the initial solid-phase Mn desorbed and 0.06% of the initial solid-phase Fe desorbed, respectively. Therefore, 0.1 M NaH_2_PO_4_ does not contribute significantly to the mineral dissolution over and above what occurs upon reaction with the O horizon leachate.

### Mineral impacts on biodegradability of aqueous DOM

**Fig. 10:**
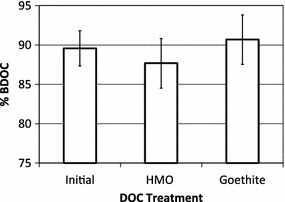
Mean % biologically degradable organic carbon (BDOC) of the initial (unreacted) dissolved DOM and the dissolved DOM after reaction with HMO and goethite at an initial C:(Mn or Fe) molar ratio of 3.1. Error bars indicated standard errors as calculated by the Tukey–Kramer HSD test

The mean BDOC expressed as a percent of the total DOC in the White Clay Creek stream water was 35 ± 4.1%—prior to injection of the O horizon leachate samples (data not shown). The native microbial population of the White Clay Creek site are able to degrade ~ 90% of the leachate DOC (Fig. 10), indicating a high biodegradability relative to the stream water DOC. Biodegradability of the leachate DOC is similar to the rates measured for a cold water extracted tulip poplar tree tissues, in which > 80% of the leachate was biodegradable both in the bioreactors and in a whole stream release [88]. Reaction with HMO or goethite at an initial C:(Mn or Fe) molar ratio of 3.1 did not statistically change the % BDOC of the aqueous DOM according to our method.

## Discussion

### Potential Mechanisms of DOM sorption to HMO and goethite

Carboxylic, phenolic, aromatic C, and polysaccharide-associated C groups comprise the principal C species types of the DOM in this study (Figs. 1, 2, 3, 4). Hydrous Mn oxide and goethite preferentially sorb carboxylic C over phenolic C and aromatic C (Fig. 3). The C–O stretching of phenolic OH peak at ~ 1265 cm^−1^ in the ATR-FTIR spectrum is maintained by HMO, but is absent in the case of goethite (Fig. 4). However, this peak is also in the range of C–O stretching of polysaccharides (Additional file 1: Table S1), and therefore may not reflect sorption of phenolic C. HMO shows stronger sorption extent for polysaccharide-associated C relative to goethite with a lower symmetric COO^−^ peak/C–O stretch of polysaccharides ratio in the ATR-FTIR spectrum (Fig. 4). Thus, polysaccharide moieties appear to play an important role in the extent and mechanism of DOM sorption by HMO, standing in contrast to findings for goethite [4, 13, 89].

The shift in the asymmetric COO^−^ peak from 1583 to 1591 cm^−1^ in the goethite-reacted DOM ATR-FTIR spectrum relative to the unreacted DOM spectrum and the associated appearance of the COO– metal stretch at 1390 cm^−1^ is evidence of partial carboxylate-metal bond formation through ligand exchange (Fig. 4), which is a well established DOM sorption mechanism for goethite [4, 13, 19, 85, 86, 90, 91]. Fourier transform infrared spectroscopy shows evidence for ligand exchange as the sorption mechanism between ferrihydrite and DOM collected from the same field site [63]. Ligand exchange is a particularly common interaction mechanism between carboxylic OH groups and metal oxide surfaces under acidic conditions, as the pKa values for most carboxylic acids in soils are between 4.3 and 4.7 [12, 85, 92]. Phenolic and aromatic C groups form complexes with metal oxides through ligand exchange under acid conditions as well [92, 93].

Reaction of goethite and HMO with the O horizon leachate resulted in consistent slight increases in pH, especially during the first few hours of reaction (data not shown). Monitoring of pH and addition of HCl was required to maintain the pH at 5.0 ± 0.2. An increase in pH is evidence of a ligand exchange reaction between DOM functional groups and hydroxyl groups at metal oxide surfaces (e.g., goethite), especially for specific adsorption of anions of weak acids [15, 94]. Nevertheless, the symmetric COO^−^ stretch peak of HMO-reacted DOM (difference spectrum between DOM sorbed onto HMO spectrum and the unreacted HMO spectrum) does not shift to the COO– metal stretch position at ~ 1390 cm^−1^ (Fig. 4), which would be indicative of ligand exchange between the carboxylate and the HMO surface. The symmetric COO^−^ stretch peak of the HMO-reacted DOM actually increases to 1414 cm^−1^ (Fig. 4), which is still in the carboxylate range [95]. Similarly, birnessite-reacted DOM (difference spectrum between DOM sorbed and unreacted birnessite) shifts wavenumber position from 1400 to 1420 cm^−1^ relative to the unreacted DOM spectrum [13]. However, the FTIR spectrum of DOM supernatant solution reacted with birnessite shows a shift of the symmetric COO^−^ stretch peak to the COO– metal stretch position of 1390 cm^−1^, consistent with the formation of Mn-carboxylate complexes in solution [13]. In this study, we did not measure ATR-FTIR spectra of DOM supernatant solutions post-reaction with HMO.

Apart from ligand exchange, another potential sorption mechanism between DOM and HMO is electrostatic interaction. However, electrostatic interaction between DOM and HMO is also unlikely at pH 5, as the PZC of HMO is 1.9, and therefore HMO surface sites should be predominantly negatively charged and electrostatically repel DOM, which also has a net negative charge [96]. Indeed, the zeta potential and electrophoretic mobility of both the unreacted DOM and unreacted HMO are negative (Table 1).

Weak interactions in various forms may contribute to sorption of DOM to HMO and goethite including physical adsorption due to favorable entropy changes, attraction of hydrophobic moieties at the exclusion of water, hydrogen bonding, and Van der Waals forces [12, 97]. However, physical adsorption is unlikely where ligand exchange occurs between DOM and metal oxides [85], as in the case of goethite. Hydrophobic interactions may occur at high DOM loadings, but are less likely where carboxylic functional groups predominate under acidic conditions [15], as in the case of the DOM in this study. The enhanced spatial correlation between C and Mn and between C and Fe with increasing C loading in DOM–HMO and DOM–goethite complexes, respectively (Additional file 1: Figure S7), as well as the lack of discrete C phases, does not support the agglomeration of hydrophobic C moieties (Fig. 5). Hydrogen bonding and Van der Waals forces cannot be excluded, but typically increase in sorption contribution for uncharged C moieties [12], which does not apply to the negatively charged DOM of the current study (Table 1). In sum, whereas ligand exchange with carboxylate groups is evidently the predominant DOM sorption mechanism for goethite, the DOM sorption mechanism on the HMO surface remains less defined, though carboxylates and polysaccharides appear to be involved (Figs. 1 and 4).

### Potential mechanisms for O horizon leachate-induced Mn reduction of HMO

At low C loadings, DOM sorbed onto HMO has a greater percent carbon signal of reduced (C–C) C species compared to DOM–goethite complexes (Figs. 1, 2). Increasing C loading on HMO clearly shows a decrease in the percent carbon signal of reduced (C–C) C and a concomitant increase in more oxidized forms (i.e., C–O/C–N and C=O) (Fig. 1). The increase in C oxidation state of DOM induced by reaction with HMO is accompanied by a reduction of Mn (Figs. 7a and 8). Increasing Mn(II) production is most strongly correlated with an increase in oxidized C (C=O) species sorbed to HMO (Additional file 1: Table S3), suggesting the potential for C oxidation and/or selective sorption of oxidized C species.

Dissolved organic matter may serve as a Mn oxide reductant through surface complexation [13, 98, 99], resulting in partial dissolution of HMO, though the DOM specific functional groups that would be involved are not clear. On the other hand, DOM has a lower capacity to induce reduction of goethite (Figs. 7b and 9) similar to results of a previous study [13]. Dissolved organic matter induces Mn reduction of birnessite, a more crystalline δ-MnO_2_ than HMO, which also has a greater capacity to oxidize DOM than does goethite through more favorable energetics [13]. The oxidative capacity of birnessite is implicated as the reason for enhanced decomposition rates of noncellulosic polysaccharides in beech litter, whereas Fe and Al oxides decrease litter decomposition rates [23]. Birnessite increases the C oxidation state of lignin in beech litter to a greater extent than does akageneite (β-FeOOH) [25]. Whether or not the greater oxidative capacity of Mn oxides over Fe oxides translates into increased litter or DOM decomposition rates will depend on chemistry of the organic C substrate and the microbial community present among other factors [23–25].

In addition to DOM, dissolved Mn(II) from the O horizon leachate is a second potential Mn reductant responsible for the dissolution of HMO. The observed reduced Mn species on the solid phase are not exclusively sorbed Mn(II) from the O horizon leachate, as reduced Mn is accompanied by HMO dissolution (Additional file 1: Figure S5) and is detected by transmission-based STXM–NEXAFS (Fig. 7a and Additional file 1: Table S2), which is a bulk species characterization technique [100]. The contribution of sorbed Mn(II) to the Mn L-edge NEXAFS signal is determined by dividing the surface thickness (~ 3 nm) by the mean particle diameter of HMO (309 nm; Table 1), which is < 1% of the total signal. Thus, the O horizon leachate not only reductively dissolved a fraction of the HMO, but also induced Mn reduction in the residual HMO. Manganese(II)-induced reductive dissolution of HMO at pH 5 does not cause a Mn speciation change of the residual HMO [101]. Therefore, the observed Mn reduction of the residual HMO in our system implicates DOM as the more probable reductant of HMO. Further work remains to discern the relative contributions of DOM and Mn(II) to the reductive dissolution of HMO. Overall, the differential DOM sorption behavior of HMO and goethite, and the exhibited differences in mineral stability in the presence of DOM and Mn(II) in the O horizon leachate may have implications for DOM partitioning and lability in forest soils.

### Extent of DOM sorption, desorption, and biodegradability

Sorption and desorption of DOM regulate the availability of organic C for microbial decomposition into assimilable substrates and ultimately into CO_2_ [3]. Extent and reversibility of DOM retention by minerals is therefore of great importance for soil C cycling. Here we show differential DOM sorption extent for HMO and goethite depending on the DOM concentration present. Goethite exhibits stronger sorption—and reaches saturation—of DOM at lower initial C:(Mn or Fe) molar ratios than does HMO, and HMO has a greater maximum DOM sorption capacity (88 ± 1 mg C g^−1^ versus 67 ± 1 mg C g^−1^) (Figs. 6 and Additional file 1: Figure S8). Goethite has a strong affinity for carboxylic C and select polysaccharide-associated functional groups at low initial C:Fe molar ratios (Fig. 2c), whereas HMO has a sustained increase in sorption of polysaccharide-associated C over a wider range of initial C:metal molar ratio (Fig. 1c).

Differential DOM sorption behavior cannot be attributed to initial SSA in this case, as the HMO and goethite tested have virtually the same SSA (138–140 m^2^ g^−1^) (Table 1). The SSA-normalized DOM sorption maxima for HMO and goethite are 6.4 × 10^2^ and 4.8 × 10^2^ μg C m^−2^, respectively (Fig. 6). Reported values for DOM sorption onto goethite (N_2_-BET SSA = 47–73 m^2^ g^−1^) at pH 4 range from 2 × 10^2^ to 1.9 × 10^3^ μg C m^−2^ depending on the chemical composition of the DOM [4, 13, 89, 102]. A more crystalline goethite (N_2_-BET SSA = 50.1 ± 0.1 m^2^ g^−1^) than that used in our study more strongly sorbed oak-derived DOM than did a more crystalline δ-MnO_2_ (birnessite; N_2_-BET SSA = 83.8 ± 0.7 m^2^ g^−1^) at all DOM concentrations tested at pH 4 [13], making DOM sorption inversely related to the initial mineral SSA in this case.

Nevertheless, applying the N_2_-BET method, which measures external SSA only, to the DOM-mineral sorption complexes helps to explain differential DOM sorption behavior by HMO and goethite (Figs. 6a and Additional file 1: Figure S9). Over an initial C:Fe molar ratio of 0–0.92, DOM sorption onto goethite increases sharply, coinciding with a sharp decrease in N_2_-BET SSA, whereas both DOM sorption and N_2_-BET SSA remain relatively constant at higher initial C:Fe molar ratios (Figs. 6a and Additional file 1: Figure S9). Thus, DOM appears to saturate and decrease the available surface area at a low C loading. In contrast, increasing DOM sorption does not have a clear impact on N_2_-BET SSA over the corresponding initial C:Mn molar ratio of 0–0.92 (Figs. 6a and Additional file 1: Figure S9). Thus, internal surfaces of HMO are evidently contributing to DOM sorption, as has been observed for As(III) sorption [61, 75], and attenuating the decrease in N_2_-BET SSA that is observed for goethite.

Ferrihydrite, a poorly crystalline Fe oxide, has a greater SSA (280 m^2^ g^−1^) than goethite, HMO, and birnessite, and has greater maximum capacity to sorb DOM extracted from the same Stroud Water Research Center site (7.2 × 10^2^ μg C m^−2^ at pH 7 and 8.5 × 10^2^ μg C m^−2^ at pH 4) [63]. Reported values for DOM sorption onto ferrihydrite at pH 4–4.6 range from 5.1 × 10^2^ to 1.1 × 10^3^ μg C m^−2^ [102, 103]. Overall, relative contributions of Mn oxides and Fe oxides to DOM sorption in soils will depend on several factors including the relative abundance of the specific phases present, the DOM concentration and chemical composition, as well as pH. Under acidic conditions, we may expect sorption extent to follow the following mineral hierarchy for O horizon extracted DOM: ferrihydrite > (HMO, goethite) > birnessite, where HMO increases in contribution to DOM sorption relative to goethite in environments with higher DOM concentrations.

Indeed, the concentration of DOM sorbed onto the solid-phase plays an important role in the extent of C desorption as well. In the case of HMO, % C desorption is lower for a C loading significantly below the sorption maximum compared to % C desorption at C loadings at or near the sorption maximum (Fig. 6). In other words, HMO binds DOM more strongly at low C loadings, probably due to ample available binding sites. Likewise, increasing sorbed DOM concentrations on ferrihydrite leads to an increase in the % C desorption at pH 4 and pH 7, potentially due to a relative increase in association of DOM with ferrihydrite pores at lower C:Fe ratios and/or the relative increase in bonding between DOM carboxyl groups and the ferrihydrite surface [63].

For the C loading range tested, we do not observe significant changes in % C desorption from goethite. For instance, increasing C loading onto goethite from 2.1 × 10^2^ to 4.8 × 10^2^ μg C m^−2^ does not significantly change the % C desorption within the error of our measurements (Fig. 6). However, in another study, about 60% C desorption by 0.1 M NaH_2_PO_4_ is observed for goethite at a lower C loading (3 × 10^1^ μg C m^−2^) [15], and decreases to < 30% C desorbed at higher DOM loadings (9 × 10^2^–1.9 × 10^3^ μg C m^−2^) [4]. Decreasing % C desorption with increasing DOM loadings may result from enhanced repulsion of the competing H_2_PO_4_^−^ by nonbinding ligands, preferential binding of strongly sorbing DOM moieties, and/or the increased concentration of metal cations capable of forming metal bridges between the DOM and mineral surfaces [15, 104, 105].

At a comparable lower C loading range (1.9 × 10^2^–2.1 × 10^2^ C μg m^−2^), the reversibility of DOM sorption is greater for goethite than for HMO. At a comparable higher C loading (4 × 10^2^ μg C m^−2^), the reversibility of DOM sorption onto HMO and onto goethite is not significantly different in the presence of 0.1 M NaH_2_PO_4_ (Fig. 6). Therefore, the chemical lability of HMO-sorbed DOM is lower than that of goethite-sorbed DOM at low C loadings in the presence of H_2_PO_4_^−^, but is similar at higher C loadings, though DOM lability in this high electrolyte solution may or may not accurately reflect lability in natural soil porewater. Importantly, the 0.1 M NaH_2_PO_4_ extraction assesses the chemical lability of the sorbed DOM remaining at the end of the 24 h sorption study, and does not address the lability of the sorbed DOM that may have been released by the HMO reductive dissolution process.

Increased desorption of DOM in the form of extracellular polymeric substances (EPS) from EPS–Al(OH)_3_ complexes correlates with an increase in biodegradation of EPS associated with Al(OH)_3_, suggesting that desorption enhances microbial utilization of DOM [106]. Thus, the efficacy that Fe and Al oxides show in protecting DOM against microbial decomposition [8, 18, 22] may extend to Mn oxides. The impact of HMO on the biodegradation of sorbed DOM has not been tested to the best of our knowledge. However, we show that the DOM remaining in solution after DOM sorption onto HMO and goethite has reached steady state (i.e., the DOM solution remaining after the 24 h DOM sorption experiment) is as biodegradable as unreacted DOM (Fig. 10). Thus, any chemical fractionation that HMO and goethite exert on DOM does not impact the biodegradability of DOM in the solution phase. Any significant impact that HMO and goethite have on DOM protection against microbial decomposition evidently would be limited to sorbed DOM. The relative impacts of HMO and goethite of biodegradability of sorbed DOM warrant future study.

## Conclusion

Manganese cycling plays a central role in fungi-promoted oxidation of O horizon material through the first several years of decomposition, after which time the Mn partitions to Mn oxides [24]. We show that reaction with O horizon leachate drives significant Mn reduction of HMO, a Mn oxide similar to biogenic Mn oxides. Manganese reduction of HMO may be driven by DOM and/or Mn(II) in the leachate. However, the observed Mn reduction of the residual HMO suggests that DOM is the more probable reductant over Mn(II) [101]. Whereas Fe and Al oxides appear to protect DOM from microbial decomposition through sorption or aqueous complex formation [8, 18, 19], the greater susceptibility to dissolution of HMO in the presence of O horizon leachate—whether due to DOM and/or aqueous Mn(II)—suggests Mn oxides may not be a long term protector of organic C in near-surface forest soils. Dissolved organic matter-induced Mn oxide dissolution may promote repartitioning of DOM into the aqueous phase, increasing the vulnerability of DOM to microbial attack relative that sorbed to minerals surfaces. Nevertheless—and contrary to our hypothesis—we show that residual HMO after partial reductive dissolution has a stronger maximum DOM sorption capacity than that of goethite. In contrast, birnessite, a common Mn oxide in soils [107], has a weak sorption capacity for DOM relative to goethite [13]. Further, at a low C loading (2 × 10^2^ μg m^−2^), DOM sorption is less reversible on HMO relative to goethite. Taken together, these observations suggest some Mn oxide phases may have a stronger capacity to regulate C partitioning in soils than previously recognized.

Much of the research on DOM sorption to mineral surfaces has been conducted using humic and fulvic acids. Previous work shows that water-extracted natural DOM, as in our O horizon leachate, contains 56% acidic humic substances—92% of which is fulvic acid with the remaining 8% being humic acid [86]. Both natural DOM and fulvic acid (Suwannee River standard) adsorb to goethite through ligand exchange with carboxylic acid groups, as we observed with DOM in this study [85]. Thus, the DOM in this study and fulvic acids may have partially overlapping chemical signatures and similar sorption behavior on metal oxides, though the biodegradability of the two DOM forms may be distinct [3]. Contrary to our hypothesis, we show that 24 h reaction with HMO does not enhance the biodegradability of DOM in the dissolved phase relative to unreacted DOM. Overall, the net ecosystem control that secondary minerals exert on organic C partitioning will be a function of the specific minerals present and warrants further exploration.

## Additional file

**Additional file 1.** Figures and Tables.

## Abbreviations

Al: aluminum
ATR-FTIR: attenuated total reflectance-Fourier transform infrared spectroscopy
BDOC: biodegradable dissolved organic carbon
BET: Brunauer–Emmett–Teller
C: carbon
DOC: dissolved organic carbon
DOM: dissolved organic matter
Fe: iron
HMO: hydrous manganese oxide
ICP–OES: inductively coupled plasma–optical emission spectroscopy
Mn: manganese
PZC: point of zero charge
STXM–NEXAFS: scanning transmission X-ray microscopy–near-edge X-ray absorption fine structure spectroscopy
XPS: X-ray photoelectron spectroscopy
XRD: X-ray diffraction
