# Supplementary material for: Impacts of hydrous manganese oxide on the retention and lability of dissolved organic matter
Source: Geochem Trans. 2018 Feb 13;19:6. doi: 10.1186/s12932-018-0051-x (PMC5811416; doi:10.1186/s12932-018-0051-x)
Supplement: Supplementary file 1 — Additional file 1. Figures and Tables. [file 12932_2018_51_MOESM1_ESM.docx]

**Additional Material**

***Impacts of hydrous manganese oxide on the retention and lability of dissolved organic matter***

Jason W. Stuckey^1,2*^, Christopher Goodwin^3^, Jian Wang^4^, Louis A. Kaplan^5,6^, Prian Vidal-Esquivel^2^, Thomas P. Beebe, Jr.^3^, Donald L. Sparks^2^

*^1^Biology Department, Multnomah University, Portland, OR 97220*

*^2^Department of Plant & Soil Sciences and Delaware Environmental Institute*

*University of Delaware, Newark, DE 19716*

*^3^Department of Chemistry and Biochemistry*

*University of Delaware, Newark, DE 19716*

*^4^Canadian Light Source Inc., University of Saskatchewan, Saskatoon, Saskatchewan S7N 2V3, Canada*

*^5^Stroud Water Research Center, 970 Spencer Road, Avondale, PA 19311*

*^6^Delaware Environmental Institute, University of Delaware, Newark, DE 19716*

**Corresponding author*

*E-mail address: jstuckey@multnomah.edu (J. W. Stuckey).*

**Biodegradable dissolved organic carbon experiment method**

Briefly, the bioreactors were constructed of paired borosilicate chromatography columns (25 mm diameter x 600 mm length; total volume of 589 ml) with polyethylene bed supports (Kontes Chromaflex, Kimble Chase, Rockwood, TN) filled with open-pored sintered borosilicate glass beads (Schott North America, Inc., Louisville, KY). The surface to volume ratio was 90,000:1. Columns were kept in the dark and continually supplied with White Clay Creek stream water in an upflow, once through mode at 4 mL min^-1^, using a peristaltic pump and Pharmed tubing (Masterflex, Cole-Parmer, Vernon Hills, IL). Teflon tubing was used to connect the paired columns and for the outflow. Full colonization of the bioreactors took approximately 6 mos. (1), and the bioreactors used in this experiment have been operating continuously for several years and are used to measure BDOC concentrations in stream water. Unamended stream water from White Clay Creek had a DOC concentration of ~ 1.5 mg C L^-1^, and was spiked with the leachate samples to final influent concentrations of ~ 2 mg C L^-1^. A final influent concentration of 2 mg C L^-1^ was targeted to avoid breakthrough of DOC by challenging the bioreactors with concentrations beyond their normal exposure range (1). The BDOC concentrations were operationally defined as the difference between the DOC concentrations of the influent and effluent waters of the bioreactors (2). The BDOC of the unamended stream water was determined prior to introduction of the leachate samples, and was subtracted from the leachate-spiked stream water BDOC measurement to provide the BDOC of the leachate samples. The bioreactors had an empty bed contact time (equal to the volume of the reactor (600 ml) divided by the flow rate) of 150 min, and three bioreactor bed volumes were allowed to pass to waste before influent and effluent waters were collected for DOC analysis. This ensured that the BDOC measurements were made on the sample being loaded onto the bioreactors (2). Additionally, by comparing the BDOC concentrations of stream water measured in the 600 ml volume bioreactors to those measured in a 1000 ml volume bioreactor, we are able to state that the 600 ml bioreactors provide an accurate measurement of the total BDOC present (3). The DOC concentrations were measured by UV-catalyzed persulfate oxidation with conductimetric detection and a detection limit of 10 μg C/L (Sievers 900 analyzer, Boulder, CO, USA). Determination of BDOC was done in triplicate for both post-HMO reacted leachate and post-goethite reacted leachate, and was repeated six times for the initial (pre-reacted) leachate. Analysis of variance and Tukey-Kramer HSD computations were performed using JMP® Pro, Version 12. SAS Institute Inc., Cary, NC, 1989-2007.

| **Table S1** Peak assignment for ATR-FTIR spectra of unreacted NOM and NOM sorbed to minerals. | | |
| --- | --- | --- |
| Peak position (cm^-1^) | Assignment | Reference |
| 1660−1642 | C=O stretch of amides | Heckman et al. (2011) and references therein |
| 1592−1582 | Asymmetric COO^-^ stretch | Heckman et al. (2011), Chen et al. (2014), and references therein |
| 1456 | CH_2_ scissoring | Heckman et al. (2011) and references therein |
| 1414−1404 | Symmetric COO^-^ stretch | Heckman et al. (2011), Chen et al. (2014), Chorover and Amistadi (2001), and references therein |
| 1390 | COO−metal stretch | Chen et al. (2014), and references therein |
| 1265−1264 | C−O stretch of polysaccharides, OH deformation of COOH, C−O stretching of phenolic OH | Heckman et al. (2011), Artz et al. (2008), and references therein |
| 1120 | C−O stretch of polysaccharides | Chen et al. (2014), and references therein |
| 1051−1041 | C−O stretch of polysaccharides | Heckman et al. (2011), Chen et al. (2014), and references therein |

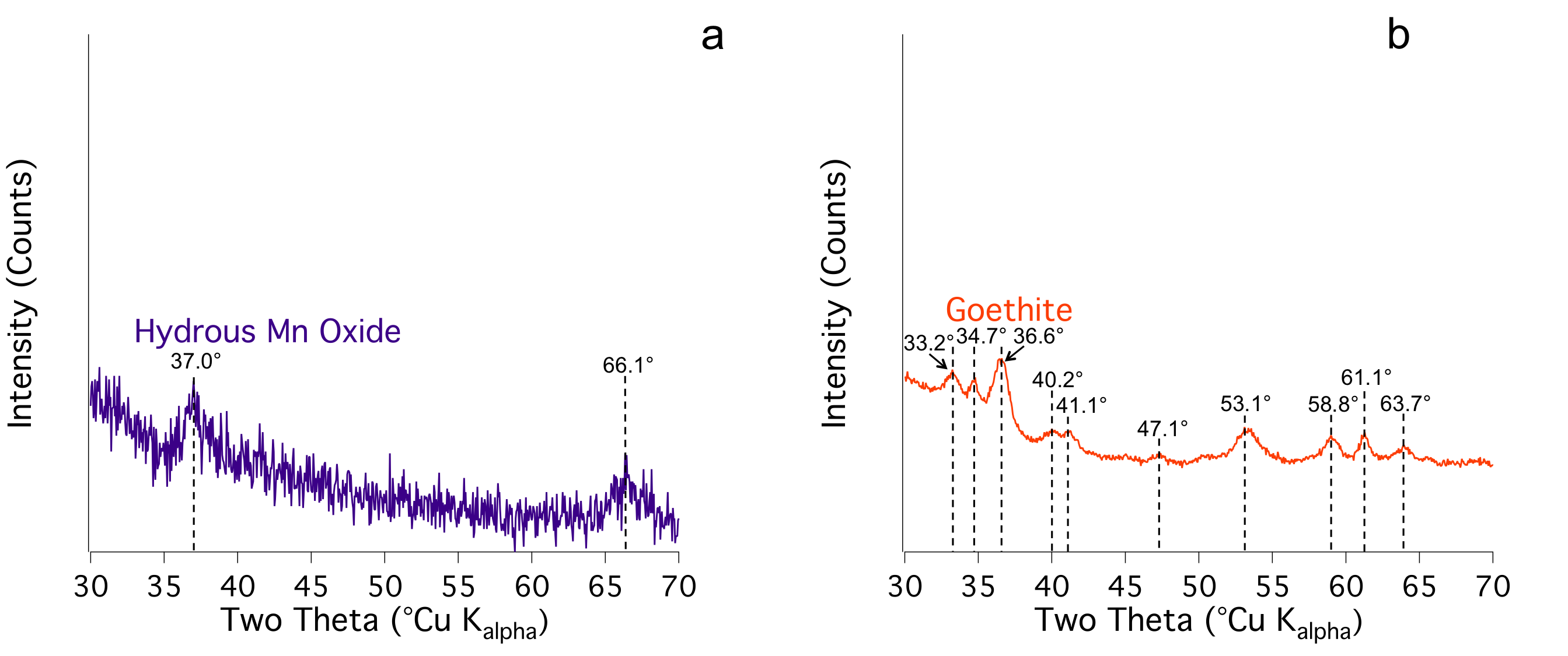


**Fig. S1** Powder X-ray diffraction (XRD) patterns for (a) HMO and (b) goethite. Reference peak positions for HMO and goethite are displayed (4, 5). The wavelength of the incident X-ray beam is 0.15418 nm (Cu K_α_). The XRD patterns are not background subtracted.

**Fig. S2** Determination of time required for batch DOM sorption experiments to reach steady state for **a** HMO and **b** goethite systems. Total C sorbed onto HMO was determined for an initial C:Mn molar ratio of 0.27. Total C sorbed onto goethite was determined for an initial C:Fe molar ratio of 0.23. Error bars indicate standard deviations of triplicates.


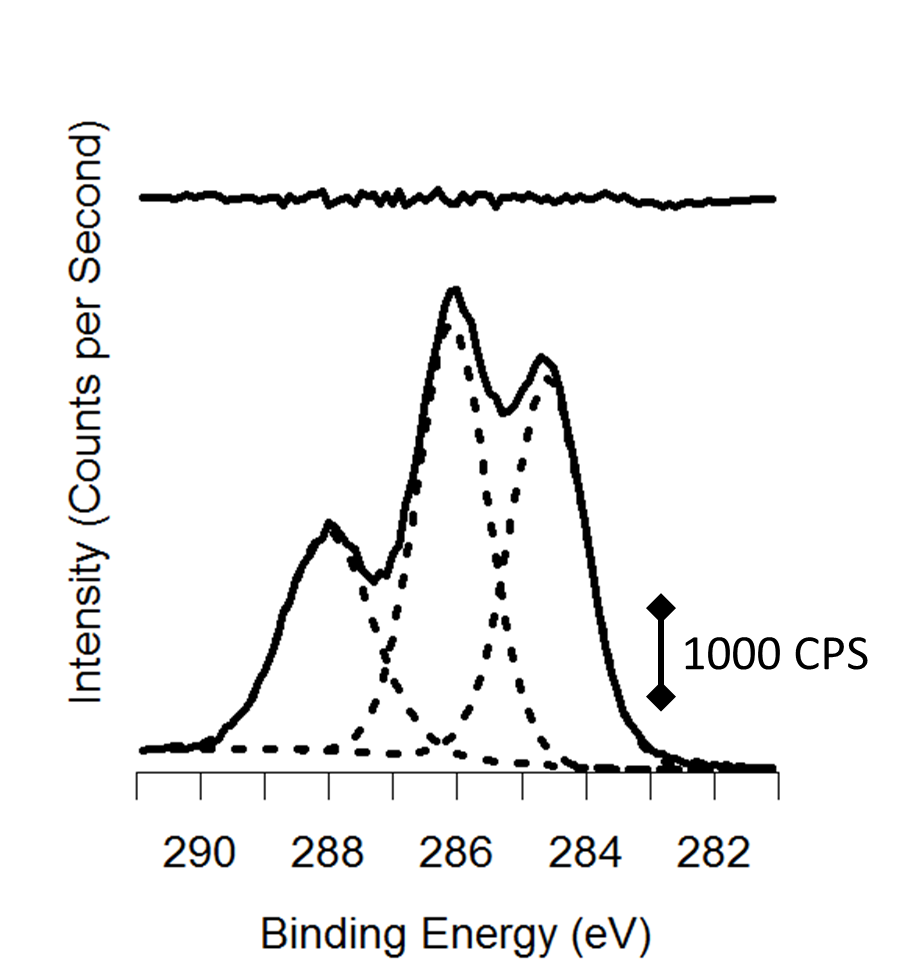


**Fig. S3** XPS fittings of the C 1s region from the DOM data; the line above the spectra is the residual from the fitted peaks shown as dashed lines. By leaving the peak positions and full width half max unconstrained the innate states can be determined rather than set introducing less bias into the fitting. A Shirley background best models the fundamental physics that changes the background shape of XPS. The fitting algorithm used Marquardt-Levenberg using a root mean squared minimum search. A Fit was deemed acceptable when the residual was not could not be distinguished from noise.


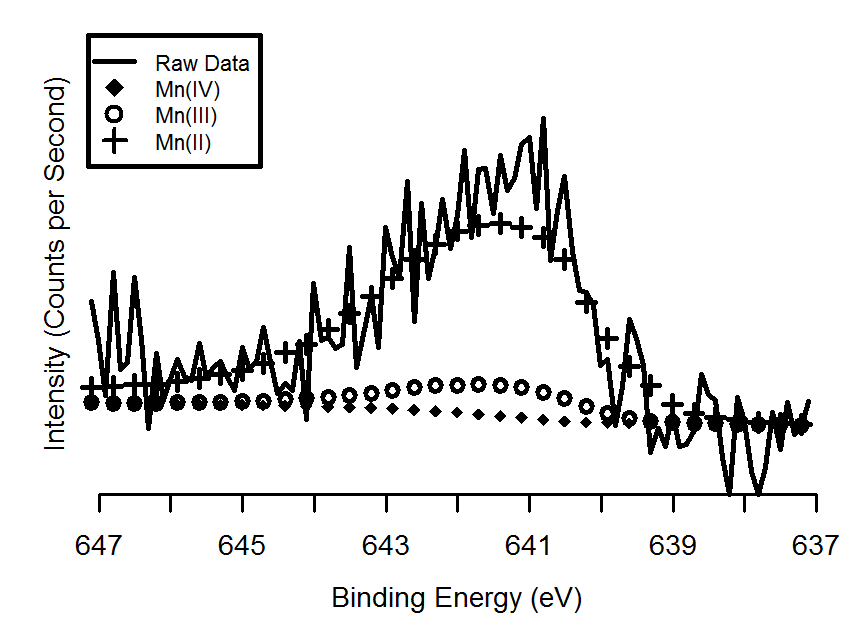


**Fig. S4** XPS of the Mn 2p 3/2 region of the Mn present in the O horizon leachate (DOM). The Mn XPS spectrum is noisy due to the low Mn concentration in the DOM, but qualitatively shows the Mn is present predominantly as Mn(II).

**Fig. S5** The net change in Mn and Fe remaining in solution after DOM sorption onto HMO and goethite reaches steady state (24 h reaction time). Data labels indicate metal and mineral system. Error bars indicate standard deviations of triplicates.

**Fig. S6** Percentage of initial solid phase Mn desorbed from HMO and percentage of initial solid phase Fe desorbed from goethite as a function of initial C:(Mn or Fe) molar ratio. Error bars indicate standard deviations of triplicates and are smaller than data points.


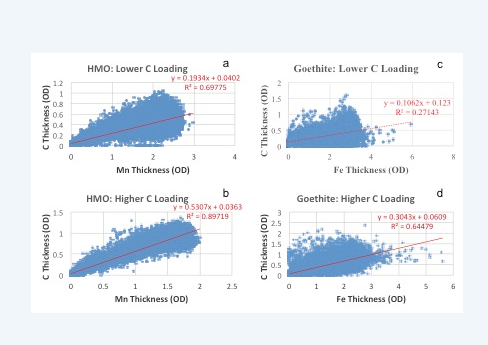


**Fig. S7** Carbon-Mn correlation plots of thickness values obtained from STXM elemental distribution maps of the thin regions (Fig. 5a-b) for the DOM-HMO sorption complexes with initial C:Mn molar ratios of **a** 0.46 and **b** 2.5. Carbon-Fe correlation plots of thickness values obtained from STXM elemental distribution maps of the thin regions (Fig. 5c-d) for the DOM-goethite sorption complexes with initial C:Fe molar ratios of **c** 0.23 and **d** 3.1.

**Fig. S8** Total C sorbed onto hydrous Mn oxide (HMO) and goethite as a function of initial C:(Mn or Fe) molar ratio in the batch system. The C:Mn molar ratio reflects the initial moles of C in DOC and moles of Mn in the HMO present. The C:Fe molar ratio reflects the initial moles of C in DOC and moles of Fe in the goethite present. Error bars indicate standard deviations of triplicates.

**Fig. S9** Specific surface area of DOM-HMO and DOM-goethite sorption complexes as measured by N_2_-BET as a function of initial C:(Mn or Fe) molar ratio.

**References**

1. Kaplan LA, Newbold JD. Measurement of streamwater biodegradable dissolved organic carbon with a plug-flow bioreactor. Water Research. 1995;29(12):2696-706.

2. Kaplan LA, Wiegner TN, Newbold J, Ostrom PH, Gandhi H. Untangling the complex issue of dissolved organic carbon uptake: a stable isotope approach. Freshwater Biology. 2008;53(5):855-64.

3. Sleighter RL, Cory RM, Kaplan LA, Abdulla HA, Hatcher PG. A coupled geochemical and biogeochemical approach to characterize the bioreactivity of dissolved organic matter from a headwater stream. Journal of Geophysical Research: Biogeosciences. 2014;119(8):1520-37.

4. Lafferty BJ, Ginder-Vogel M, Zhu MQ, Livi KJT, Sparks DL. Arsenite Oxidation by a Poorly Crystalline Manganese-Oxide. 2. Results from X-ray Absorption Spectroscopy and X-ray Diffraction. Environ Sci Technol. 2010;44(22):8467-72.

5. Nagai T, Kagi H, Yamanaka T. Variation of hydrogen bonded O··· O distances in goethite at high pressure. Am Miner. 2003;88(10):1423-7.
